# Identification of functional pathways and potential genes associated with interferon signaling during human adenovirus type 7 infection by weighted gene coexpression network analysis

**DOI:** 10.1007/s00705-023-05707-8

**Published:** 2023-04-05

**Authors:** Zhongying Yang, Jianhua Wei, Yu He, Luo Ren, Shiyi Chen, Yu Deng, Na Zang, Enmei Liu

**Affiliations:** Department of Respiratory Children’s Hospital of Chongqing Medical University, National Clinical Research Center for Child Health and Disorders, Ministry of Education Key Laboratory of Child Development and Disorders,Chongqing Key Laboratory of Child Infection and Immunity, Children’s Hospital of Chongqing Medical University, Chongqing, 400014 China

**Keywords:** Human adenovirus type 7, Coexpression network, Weighted gene coexpression network analysis (WGCNA), Pathogenesis

## Abstract

Human adenovirus type 7 (HAdV-7) can cause severe pneumonia and complications in children. However, the mechanism of pathogenesis and the genes involved remain largely unknown. We collected HAdV-7-infected and mock-infected A549 cells at 24, 48, and 72 hours postinfection (hpi) for RNA sequencing (RNA-Seq) and identified potential genes and functional pathways associated with HAdV-7 infection using weighted gene coexpression network analysis (WGCNA). Based on bioinformatics analysis, 12 coexpression modules were constructed by WGCNA, with the blue, tan, and brown modules significantly positively correlated with adenovirus infection at 24, 48, and 72 hpi, respectively. Functional enrichment analysis indicated that the blue module was mainly enriched in DNA replication and viral processes, the tan module was largely enriched in metabolic pathways and regulation of superoxide radical removal, and the brown module was predominantly enriched in regulation of cell death. qPCR was used to determine transcript abundance of some identified hub genes, and the results were consistent with those from RNA-Seq. Comprehensively analyzing hub genes and differentially expressed genes in the GSE68004 dataset, we identified *SOCS3*, *OASL*, *ISG15*, and *IFIT1* as potential candidate genes for use as biomarkers or drug targets in HAdV-7 infection. We propose a multi-target inhibition of the interferon signaling mechanism to explain the association of HAdV-7 infection with the severity of clinical consequences. This study has allowed us to construct a framework of coexpression gene modules in A549 cells infected with HAdV-7, thus providing a basis for identifying potential genes and pathways involved in adenovirus infection and for investigating the pathogenesis of adenovirus-associated diseases.

**Supplementary information:**

The online version contains supplementary material available at 10.1007/s00705-023-05707-8.

## Introduction

Human adenoviruses (HAdVs) belong to the genus *Mastadenovirus* within the family *Adenoviridae* and are classified into seven species (A-G). Since the first adenovirus was characterized [32], over 100 types have been recognized based on genome sequences (https://hadvwg.gmu.edu/). Among these, members of species B (HAdV − 3, 7, 11, 14, 16, 21, 34, 35, 50, and 55), C (HAdV − 1, 2, 5, 6, and 57), and E (HAdV-4) are known to cause a variety of acute respiratory diseases [23, 29, 41]. However, HAdV-3 and HAdV-7 are the major types responsible for lower respiratory diseases, and HAdV-7 is more likely to cause severe pneumonia and significant mortality, especially in young children [9, 37]. However, the molecular mechanisms underlying adenovirus-associated diseases remain largely unknown, and the genes and pathways involved in these processes still need to be identified.

Several studies examining RNA and protein expression profiles have revealed multiple alterations in gene expression in infected cells [45]. For example, quantitative proteomic analysis of HAdV-2-infected A549 cells and transcriptomic analysis of HAdV-2-infected human primary lung fibroblasts at 24 and 48 hours postinfection (hpi) have shown that viral infection affects the expression of genes and proteins involved in various cellular pathways [1, 44]. However, other HAdVs in different host cells using different methodologies also need to be explored to improve our understanding of adenovirus infection and adenovirus-associated diseases. RNA sequencing (RNA-Seq) for transcriptome profiling uses deep sequencing technology, with the resulting reads used to produce genome-scale transcription maps composed of both the transcription structure and expression level of each gene for a specific developmental stage or physiological condition, thus generating a tremendous amount of data [36].

Many genes are involved in the pathogenesis of adenovirus-associated diseases, thus constituting a complex network. The exploration of gene-network signatures associated with complex diseases and biological processes can be achieved by weighted gene coexpression network analysis (WGCNA) [18]. Using this algorithm, gene expression data are transformed into coexpression modules, and trait data are effectively integrated to identify functional pathways and key genes implicated in the pathogenesis process [40].

In this study, we constructed coexpression modules using expression data obtained from HAdV-7-infected A549 cells and identified modules containing highly coexpressed genes. Functional enrichment analysis was performed on the modules of interest, and hub genes were identified in the corresponding modules. Candidate genes with biomarker and therapeutic target potential were also identified. This study provides a foundation for further investigation of the pathogenesis of adenovirus-associated diseases and potential therapies.

## Materials and methods

### Cell culture and adenovirus infection

The HAdV-7 strain (CQ45_2019, MT113943) used in this study was originally isolated from nasal aspirates from a child infected with HAdV-7 infection and was stored at − 80°C. The strain was cultured in A549 cells obtained from the American Type Culture Collection (ATCC) and subsequently maintained in our lab. The HAdV-7 particles were purified by standard caesium chloride gradient centrifugation and suspended in Hank’s balanced salt solution (HBSS) with 0.5% fetal bovine serum (FBS, Gibco) as described previously [17]. The median tissue culture infectious dose (TCID_50_) of the virus was calculated as described previously [34]. A549 cells were infected with HAdV-7 at a multiplicity of infection (MOI) of 1, and three biological replicates of infected and mock-infected cells were collected at 24, 48, and 72 hpi for RNA-Seq. All virus experiments were performed in biosafety level 2 facilities following governmental and institutional guidelines.

### RNA library construction and sequencing

Total RNA was isolated and purified from six samples of HAdV-7-infected and mock-infected A549 cells (three each) using TRIzol Reagent (Invitrogen, Carlsbad, CA, USA), following the manufacturer’s procedures. Poly(A) RNA was purified from 1 µg of total RNA using Dynabeads Oligo (dT)25-61005 (Thermo Fisher, CA, USA) with two rounds of purification. The poly(A) RNA was then fragmented into small pieces using the NEBNext Magnesium RNA Fragmentation Module (cat. no. E6150, NEB, USA) at 94°C for 5–7 min. The cleaved RNA fragments were then reverse transcribed to create cDNA using SuperScript II Reverse Transcriptase (cat. no. 1896649, Invitrogen, USA) for the synthesis of U-labeled second-stranded DNA with *E. coli* DNA polymerase I (cat. no. m0209, NEB, USA), RNase H (cat. no. m0297, NEB, USA), and dUTP solution (cat. no. R0133, Thermo Fisher, USA). An A-base was then added to the blunt ends of each strand for ligation to the indexed adapters. Each adapter contained a T-base overhang to ligate the adapter to the A-tailed fragmented DNA, and single- or dual-index adapters were ligated to the fragments. After treatment of the U-labeled second-stranded DNA with heat-labile UDG enzyme (cat. no. m0280, NEB, USA), the ligated products were amplified by polymerase chain reaction (PCR). The average insert size for the final cDNA library was 300 ± 50 bp. Lastly, 2 × 150-bp paired-end sequencing (PE150) was performed using an Illumina NovaSeq 6000 instrument (LC-Bio-Technology Co., Ltd., Hangzhou, China) following the vendor’s recommended protocols.

## Bioinformatic analysis

### Correlation analysis of replicas and principal component analysis (PCA)

When examining gene expression in the samples, we performed R correlation analysis of two parallel experiments to evaluate repeatability. PCA was performed using the R package “model” (http://www.r-project.org/) to detect possible clusters or outliers among samples.

### Differentially expressed gene (DEG) screening

Genes that were differentially expressed between uninfected and adenoviral-infected cells were identified using the R package “DESeq2” [27]. Genes with a fold-change > 2 or < 0.5 and a *P*-value < 0.05 were considered DEGs.

### Construction of a coexpression network

A coexpression network based on HAdV-7-infected and mock-infected cells was constructed using the R package “WGCNA” [18]. The gradient method was applied to test the independence and average degree of connectivity of different modules with different power values (ranging from 1 to 30). We selected a soft-thresholding power of 4 when the degree of independence was 0.9. The minimum number of genes was set to 40 to ensure high reliability of results. To merge possibly similar modules, we defined 0.1 as the threshold for cut height. Coexpression modules were identified using the WGCNA algorithm, and similar modules (module eigengenes) were merged into a single module and then used for further interpretation.

### Functional enrichment analysis

The constructed modules were arranged by number of genes. To gain insight into the function of the genes in the modules most affected by HAdV-7 infection, we used the Database for Annotation, Visualization, and Integrated Discovery (DAVID) (https://david.ncifcrf.gov/home.jsp/) to perform Gene Ontology (GO) and Kyoto Encyclopedia of Genes and Genomes (KEGG) pathway enrichment analyses. A *P*-value of < 0.05 was set as the cutoff threshold, and, if there were more than 10 records, the top 10 records were extracted. The R package “ggplot” was used to show the results graphically.

### Hub gene identification and validation

Genes in each module of interest with module membership (MM) > 0.8 and gene significance (GS) > 0.2 were identified as important intramodular genes, indicating a significant correlation with HAdV-7 infection. Subsequently, the DEGs obtained by RNA-Seq were overlapped with the corresponding important intramodular genes to identify hub genes, the results of which are presented as a table and Venn diagrams (http://bioinformatics.psb.ugent.be/webtools/Venn/). To further validate the confidence of the high-throughput transcriptome sequencing, five hub genes were randomly selected for analysis via quantitative PCR (qPCR).

Total RNA was extracted from HAdV-7-infected and mock-infected A549 cells using Direct-zol RNA Miniprep (R2050, Zymo Research) and TRIzol Reagent (Takara, Kusatsu, Japan) according to the manufacturer’s instructions. Purified RNA was reverse transcribed into cDNA using a Transcriptor cDNA Synthesis Kit 2 (Roche). Newly synthesized cDNA was analyzed by qPCR using SYBR Green PCR master mix (Vazyme) on a CFX96 Touch Real-Time PCR Detection System in 20-µl reactions. Relative mRNA expression was quantified using the comparative threshold (2^−ΔΔCT^) method, with GAPDH as a calibrator. The sequences of the primers that were used are shown in Table 1.

**Table 1:** Sequences of primers used in the study

Gene	Forward primer sequence (5’→3’)	Reverse primer sequence (5’→3’)
*KIF5C*	ATCCCACGAATTGCCCATGAT	CCCTTTACATACGGGACTCTGT
*SFRP5*	CTGAGATGCTGCACTGCCACAA	GTCAGCACTGTGCTCCATCTCA
*TP73*	CACCTCAGCTCTCCATCTTATTG	GCATGGGTCTTAGCCTTTCT
*OASL*	CCATTGTGCCTGCCTACAGAG	CTTCAGCTTAGTTGGCCGATG
*SOCS3*	CATCTCTGTCGGAAGACCGTCA	GCATCGTACTGGTCCAGGAACT

### Identification of potential candidate genes

To identify the hub genes that play important roles in adenovirus infection, we selected an appropriate dataset, GSE68004, from the NCBI Gene Expression Omnibus (GEO; https://www.ncbi.nlm.nih.gov/geo/) by entering the keyword ‘HAdV’. This dataset contains blood RNA data from 76 pediatric patients with complete Kawasaki disease (KD), 13 with incomplete KD, 19 with HAdV, 17 with group A streptococcal (GAS) disease, and 37 healthy controls (HC). The GEO2R tool in the GEO database was employed to analyze samples from patients with HAdV and HC. Criteria for identifying DEGs via GEO2R analysis were set to *P* < 0.05, logFC > 1, or logFC < -1. The DEGs and hub genes in the key modules were assessed through reciprocal overlap (http://bioinformatics.psb.ugent.be/webtools/Venn/), and overlapping genes were regarded as candidate genes. An interaction network was constructed using GeneMANIA (http://genemania.org/, accessed on 5 January 2021).

### Statistical analysis

Statistical analysis of candidate gene expression was performed using GraphPad Prism 8.0 (GraphPad Software Inc.). Results are expressed as the mean ± standard error of the mean (SEM). Data were analyzed using two-way analysis of variance (ANOVA) for multiple comparisons. Differences with *P* < 0.05 were considered statistically significant.

## Results

### Transcriptomic analysis of HAdV-7-infected and mock-infected A549 cells

**Fig. 1:**
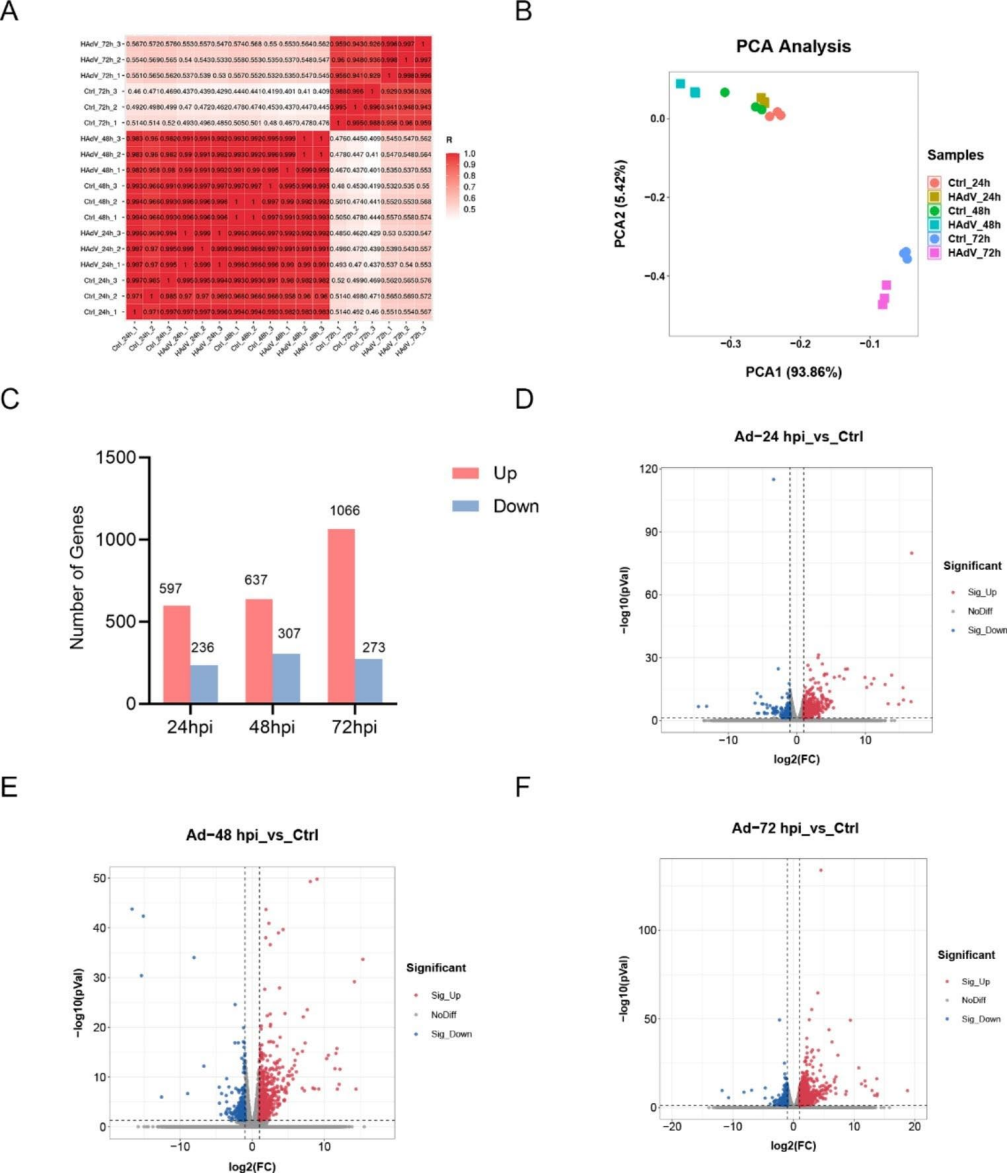
Transcriptomic analysis of HAdV-7-infected and mock-infected A549 cells. (**A**) Correlation heat map of A549 cell samples. Numbers in boxes represent Pearson correlation coefficients between two corresponding samples. (**B**) PCA of tested samples. (**C**) Numbers of DEGs between control and HAdV-7-infected cells after 24, 48, and 72 hpi. (**E**-**F**) Volcano plot of DEGs

To better understand the underlying mechanisms of HAdV-7 infection, we conducted comparative transcriptomic analysis of HAdV-7-infected and mock-infected A549 cells. Pearson correlation analysis based on sample expression was used to evaluate differences between groups. As shown in Fig. 1A, samples were closely correlated, with a minimum coefficient of 0.971 between groups, indicating good parallelism between biological replicates and reliable sequencing results. As a powerful tool for reducing dimensionality of complex datasets, PCA shows high performance in excluding outliers and artifacts [35]. Figure 1B shows a PCA plot of the 18 specimens in the two-dimensional plane. The first and second principal components (PC1 and PC2) explained 93.86% and 5.42% of sample variance, respectively. At each time point, samples from infected and uninfected (control) cells were separated, while the closeness of samples within groups showed good repeatability. Based on differential expression analysis, 834 DEGs (598 up- and 236 downregulated) at 24 hpi, 945 DEGs (638 up- and 307 downregulated) at 48 hpi, and 1399 DEGs (1066 up- and 273 downregulated) at 72 hpi were identified when comparing mock-infected and HAdV-7-infected cells (Fig. 1C). The distribution of the DEGs is shown in a volcano plot in Fig. 1D-F.

**Fig. 2:**
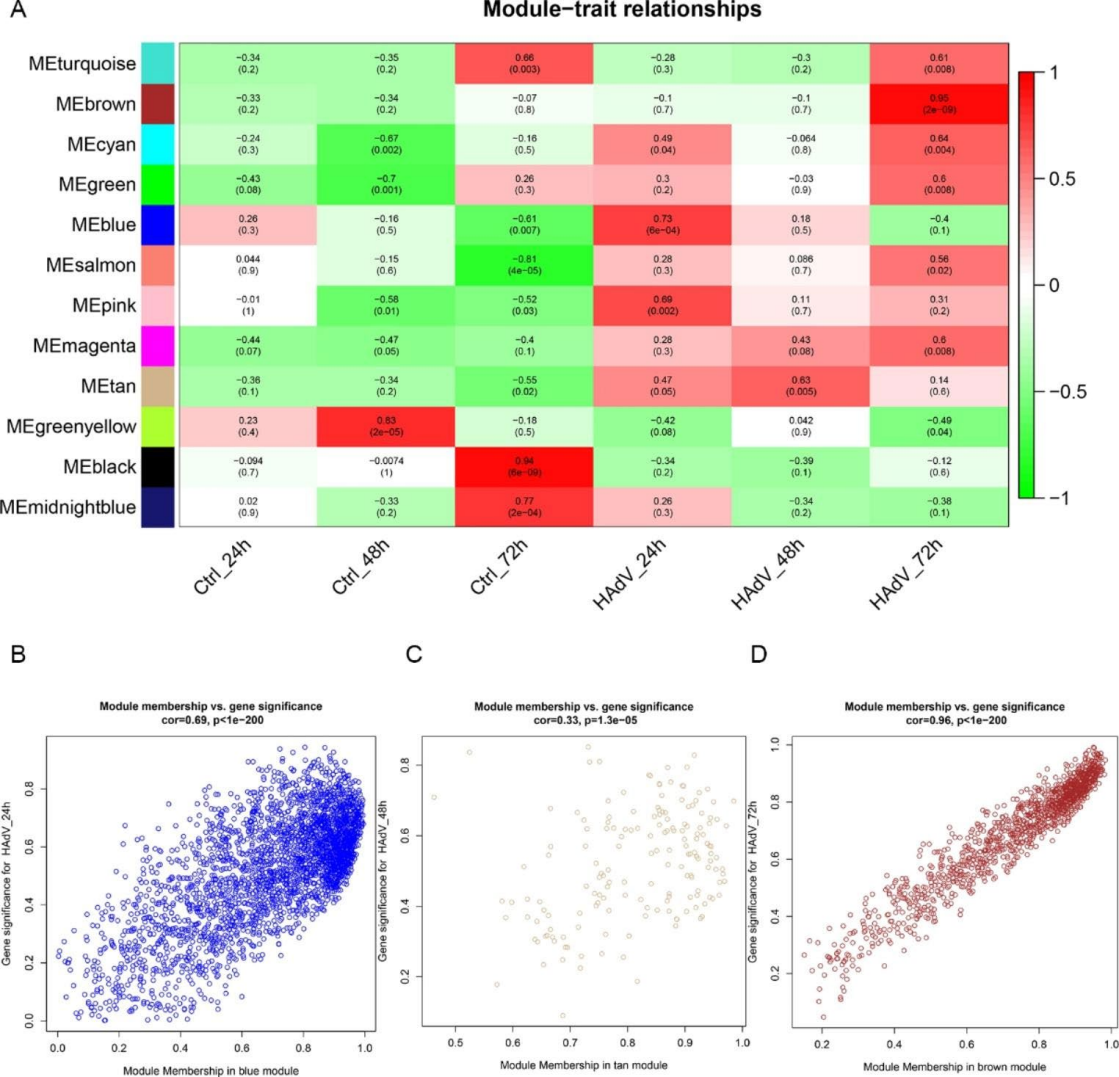
(**A**) Correlation heatmap between modules and HAdV-7 infection. Each cell contains corresponding correlation and *P*-value. The table is color-coded by correlation according to the color legend. (**B**-**D**) Scatterplot of gene significance (GS) for HAdV-7 infection vs. module membership (MM) in the blue, tan, and brown modules. One dot represents one gene, and the corresponding correlation coefficient and *P*-value are shown above the scatter plot.

### Gene coexpression modules correspond to HAdV-7 infection

Module-trait associations were analyzed by correlating module eigengenes with clinical traits (i.e., HAdV-7 infection). As shown in Fig. 2A, multiple modules were related to Ad-24 hpi, Ad-48 hpi, and Ad-72 hpi. Among them, the blue module (containing 2934 genes) was significantly associated with Ad-24 hpi (r = 0.73; *P* < 0.001), the tan module (containing 167 genes) was significantly associated with Ad-48 hpi (r = 0.63; *P* = 0.005), and the brown module (containing 1259 genes) was highly positively correlated with Ad-72 hpi (r = 0.95; *P* < 0.001). Therefore, these three modules were identified as key modules for HAdV-7 infection. We constructed a scatterplot of GS vs. MM in the key modules (Fig. 2B-D). Genes in the blue, tan, and brown modules that were most significantly associated with HAdV-7 infection characteristics (GS) were also the most important elements of the modules (MM), as demonstrated by the upper right-hand genes in the plot.

**Fig. 3:**
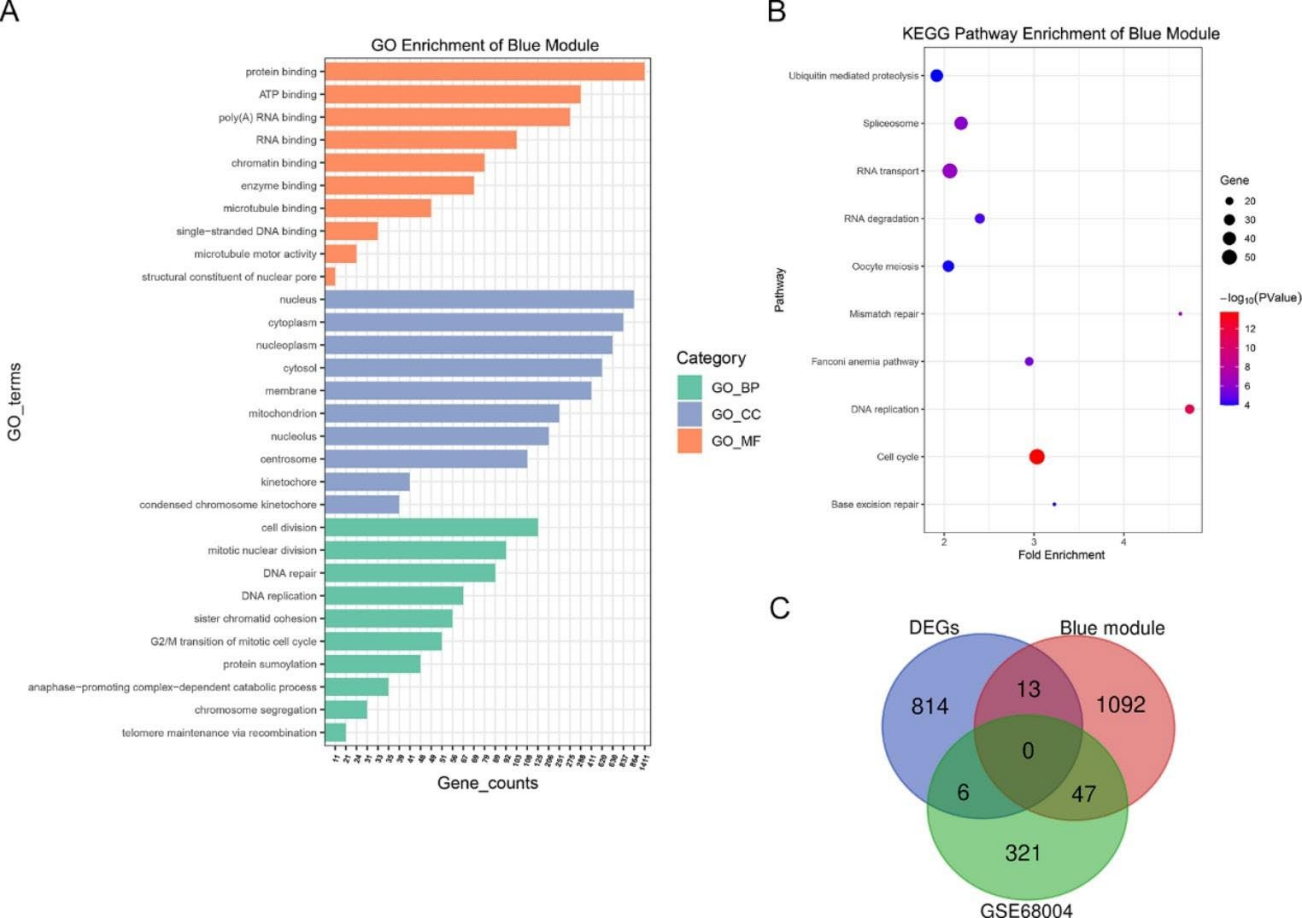
(**A**-**B**) GO and KEGG enrichment analysis of genes in the blue module. The gene count is the number of genes annotated in GO terms in a specific module. (**C**) Venn diagram of DEGs identified by RNA-Seq, genes in the blue module, and DEGs from the GSE68004 dataset.

### Functional enrichment analysis of genes in modules of interest

**Fig. 4:**
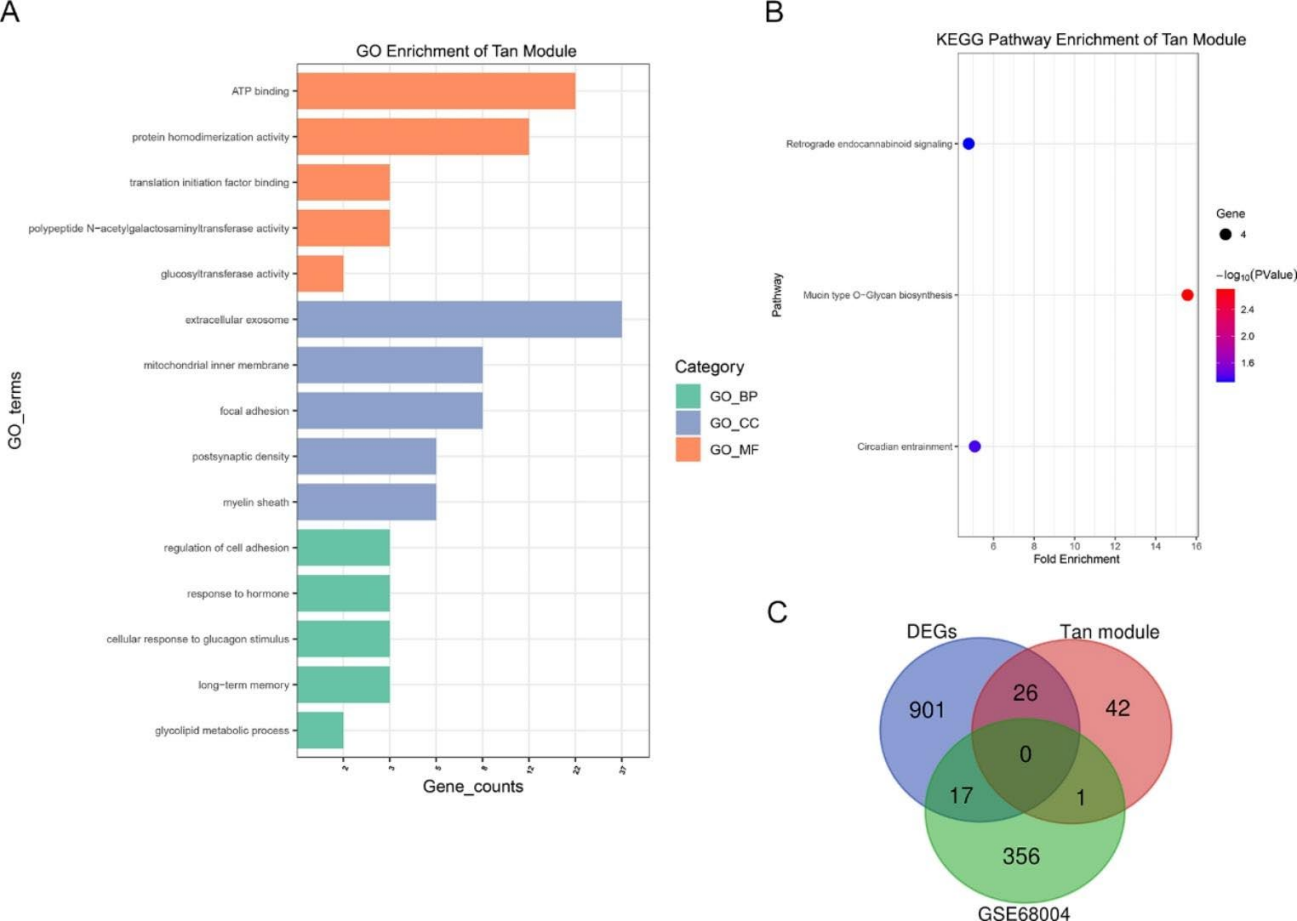
(**A**-**B**) GO and KEGG enrichment analysis of genes in the tan module. (**D**) Venn diagram of DEGs identified by RNA-Seq, genes in the tan module, and DEGs from the GSE68004 dataset.

**Fig. 5:**
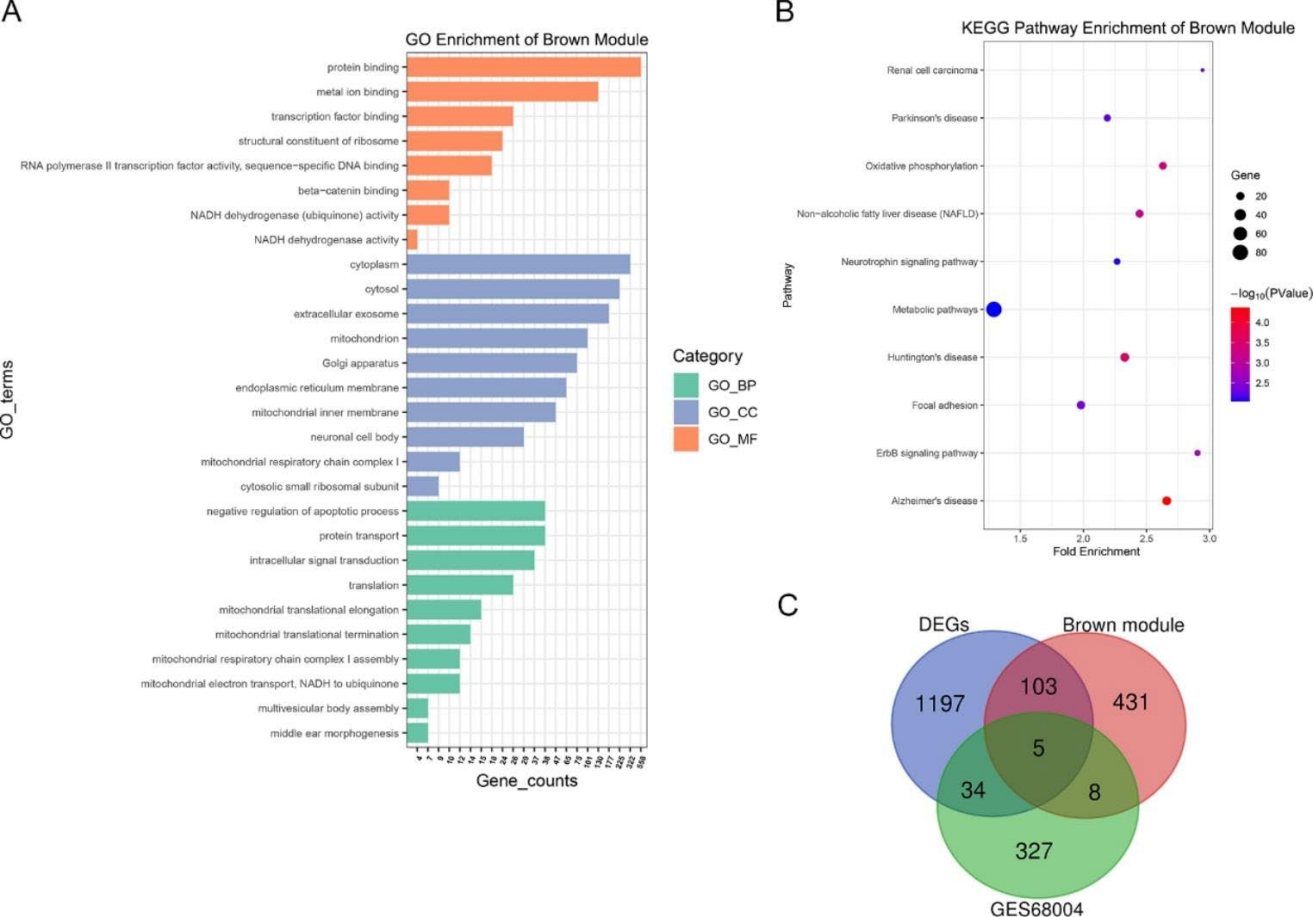
(**A**-**B**) GO and KEGG enrichment analysis of genes in the brown module. (**C**) Venn diagram of DEGs identified by RNA-Seq, genes in the brown module, and DEGs from the GSE68004 dataset.

To better understand the function of genes in key modules, GO and KEGG pathway enrichment analyses were performed, with the top terms of each category presented in Figs. 3–5. KEGG analysis demonstrated that genes in the blue module were primarily enriched in pathways related to cell cycle regulation and cell proliferation, including DNA replication, mismatch repair, RNA transport, and spliceosome function, consistent with biological process (BP) and molecular function (MF) enrichment analysis (Fig. 3A-B). Genes in the blue module associated with cell component (CC) terms were primarily enriched in those associated with the cell nucleus, including the nucleoplasm and nucleolus. Genes in the tan module were enriched in mucin type O-glycan biosynthesis, circadian entrainment, retrograde endocannabinoid signaling, and metabolic pathways. GO enrichment showed that tan module genes were significantly associated with cell metabolism, such as glycolipid metabolic process, polypeptide N-acetylgalactosaminyltransferase activity, and glucosyltransferase activity (Fig. 4A-B). Genes in the brown module were enriched in oxidative phosphorylation, the ErbB signaling pathway, and metabolic pathways. Furthermore, GO analysis indicated that genes in the brown module were primarily associated with mitochondrial respiratory chain functions, protein translation, and apoptosis, as well as cellular structure of the mitochondrial respiratory chain and protein synthesis (Fig. 5A-B).

### Screening and validation of hub genes

**Fig. 6:**
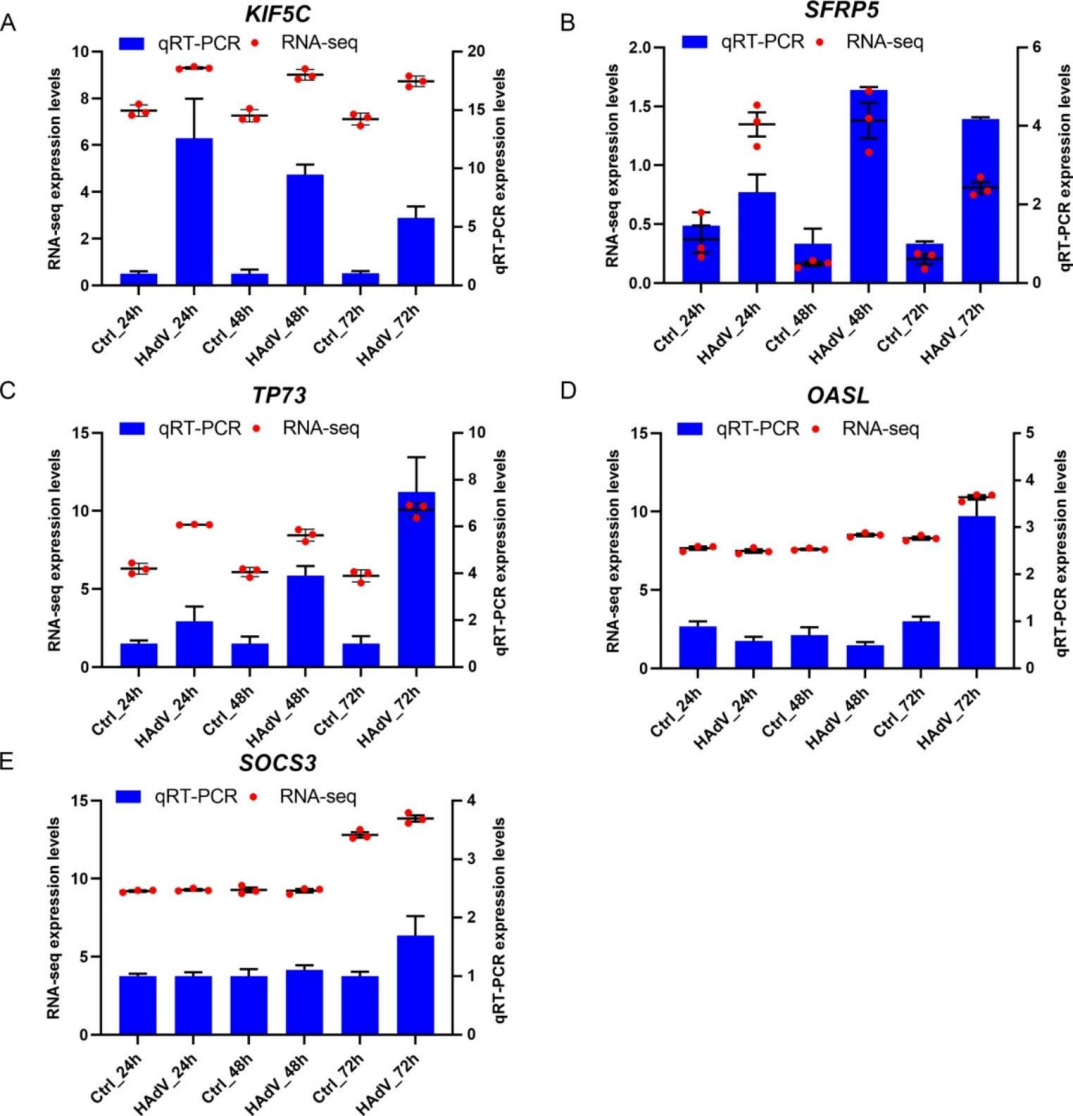
Quantitative real-time PCR verification of hub genes related to HAdV-7 infection. The left and right vertical axis scales correspond to RNA-Seq values and qRT-PCR values, respectively. Data are representative of two independent experiments (3–6 samples per group).

Important intramodular genes in the three main modules are shown in the upper right section of Fig. 2B-D, suggesting that they are not only functionally associated with HAdV-7 infection but also significantly associated with its characteristic traits. Furthermore, genes in the blue, tan, and brown modules overlapped with DEGs based on the RNA-Seq results, with the intersecting genes defined as hub genes (Fig. 3-5C). These genes had high intra-module connectivity and exhibited significant differences in expression (Supplementary Table S1). Of the genes in the blue module, two are involved in the positive regulation of the *ERK1* and *ERK2* cascade, including *TNFAIP8L3*, which belongs to the tumor necrosis factor-alpha (TNF-α)-induced protein 8 (TNFAIP8/TIPE) family and participates in the regulation of inflammatory responses, immune homeostasis, and the proliferation/apoptosis axis by regulating *PI3K* and downstream mediators, including nuclear factor-κB (NF-κB), mitogen-activated protein kinases (MAPKs; e.g., *ERK-1* and *ERK-2*, *JNK*, and *p38*), and interferon-regulatory factors (IRFs; e.g., *IRF3* and *IRF7*) [6, 11, 12]. Among the 26 hub genes in the tan module, *PIK3AP1* and *ARID5A* are related to the inflammatory response and innate immune system, and *KIF5C* and *PID1* are associated with energy and metabolism, indicating that energy production and metabolism may play an important role in HAdV-7 infection. Moreover, the presence of *SFRP5* and *TCF7* in the tan module suggests that the WNT signaling pathway may be involved in adenovirus infection. Of the 103 hub genes in the brown module, many participate in the regulation of transcription and cell death. Importantly, *TP73*, *JUND*, *JUNB*, *FOSB*, and *BATF3* are involved in crosstalk among multiple pathways. Five hub genes were selected for qPCR to confirm the differential expression levels determined by RNA-Seq. Comparing transcriptome data with qPCR results, we found that the patterns of gene transcript abundance were consistent (Fig. 6A-E), confirming not only the reliability of our transcriptome data but also the network centrality and potential roles of key genes in HAdV-7 infection.

### Identification of representative adenovirus-infection-related candidate genes

We also explored the relationship between expression profile changes in adenovirus-infected cells and clinical samples. Differential gene analysis was performed on the GSE68004 dataset of the HAdV and HC groups, resulting in 374 DEGs. Candidate genes were selected based on the intersection of significant genes and DEGs from the GSE68004 dataset (Fig. 3-5C). Only the brown module contained genes at the intersection of the three different categories, including suppressor of cytokine signaling (*SOCS3*), oligoadenylate synthetases-like (*OASL*), interferon (IFN)-stimulated gene product 15 (*ISG15*), IFN-induced protein with tetratricopeptide repeats 1 (*IFIT1*), and hemoglobin subunit alpha 1 (*HBA1*).

### Possible interactions between adenovirus and host cells

**Fig. 7:**
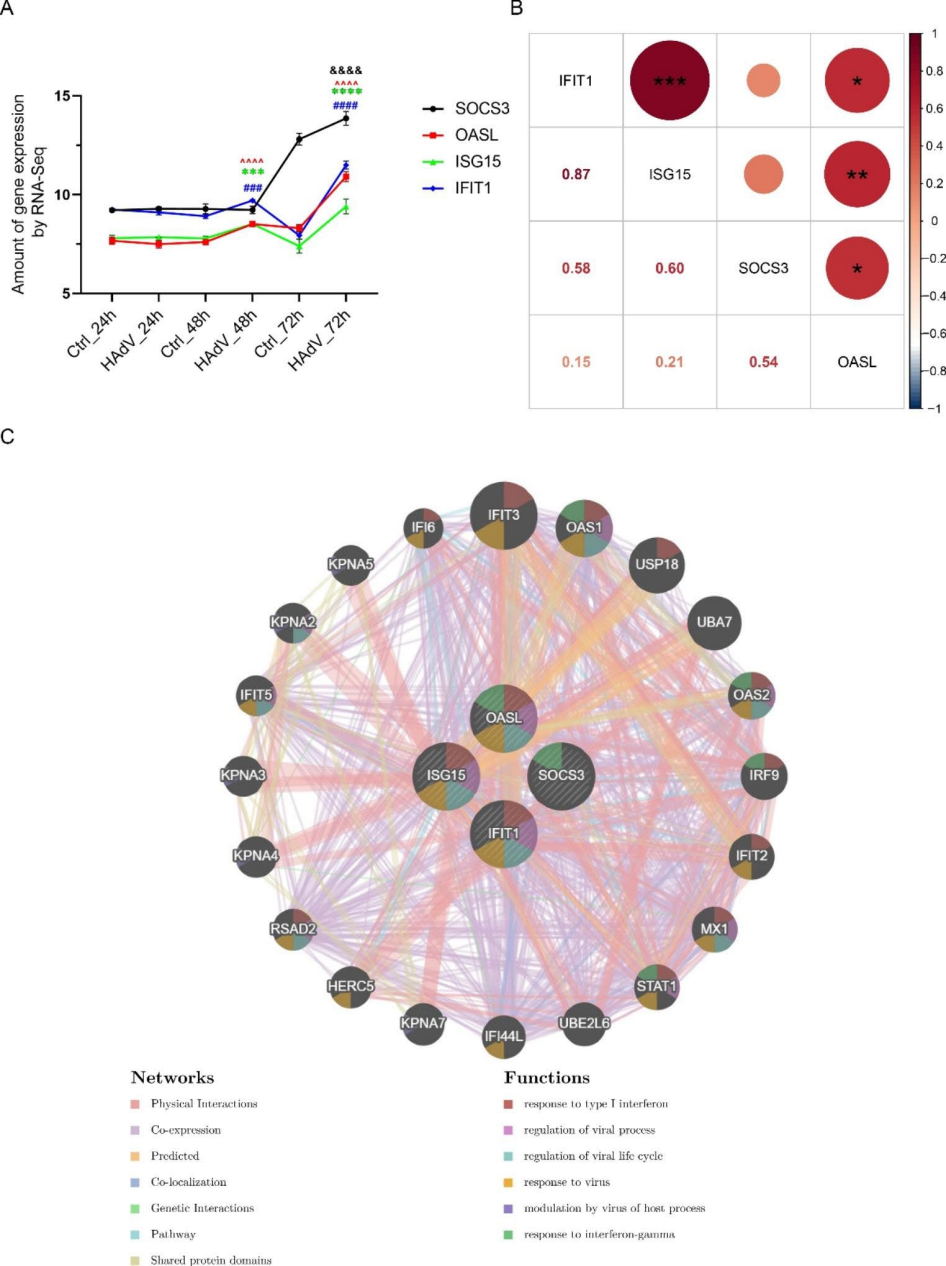
(**A**) Gene expression level shown by RNA-Seq graphs at three time points. Data are representative of two independent experiments performed on three samples per group. &, *P* < 0.05, &&, *P* < 0.01, &&&, *P* < 0.001, &&&&, *P* < 0.0001, HAdV-7-infected vs. control cells for SOCS3; *, *P* < 0.05, **, *P* < 0.01, ***, *P* < 0.001, ****, *P* < 0.0001, HAdV-7-infected vs. control cells for ISG15; ^, *P* < 0.05, ^^, *P* < 0.01, ^^^, *P* < 0.001, ^^^^, *P* < 0.0001, HAdV-7-infected vs. control cells for OASL; #, *P* < 0.05, ##, *P* < 0.01, ###, *P* < 0.001, ####, *P* < 0.0001, HAdV-7-infected vs. control cells for IFIT1. (**B**) Correlation between the candidate genes according to gene expression. Correlation coefficients (r, Spearman rank correlation) are shown at the lower left, and significance is shown at the upper right (*, *p* < 0.05, **, *p* < 0.01, *** *p* < 0.001). (**C**) Gene-to-gene interaction network of the four candidate genes in the GeneMANIA dataset, wherein the size of each node indicates the strength of interaction, the color of lines represents the types of interactions between genes (color code in “Networks” legend), and colored node circle sections show the functions of the respective genes (color code in “Functions” legend).

**Fig. 8:**
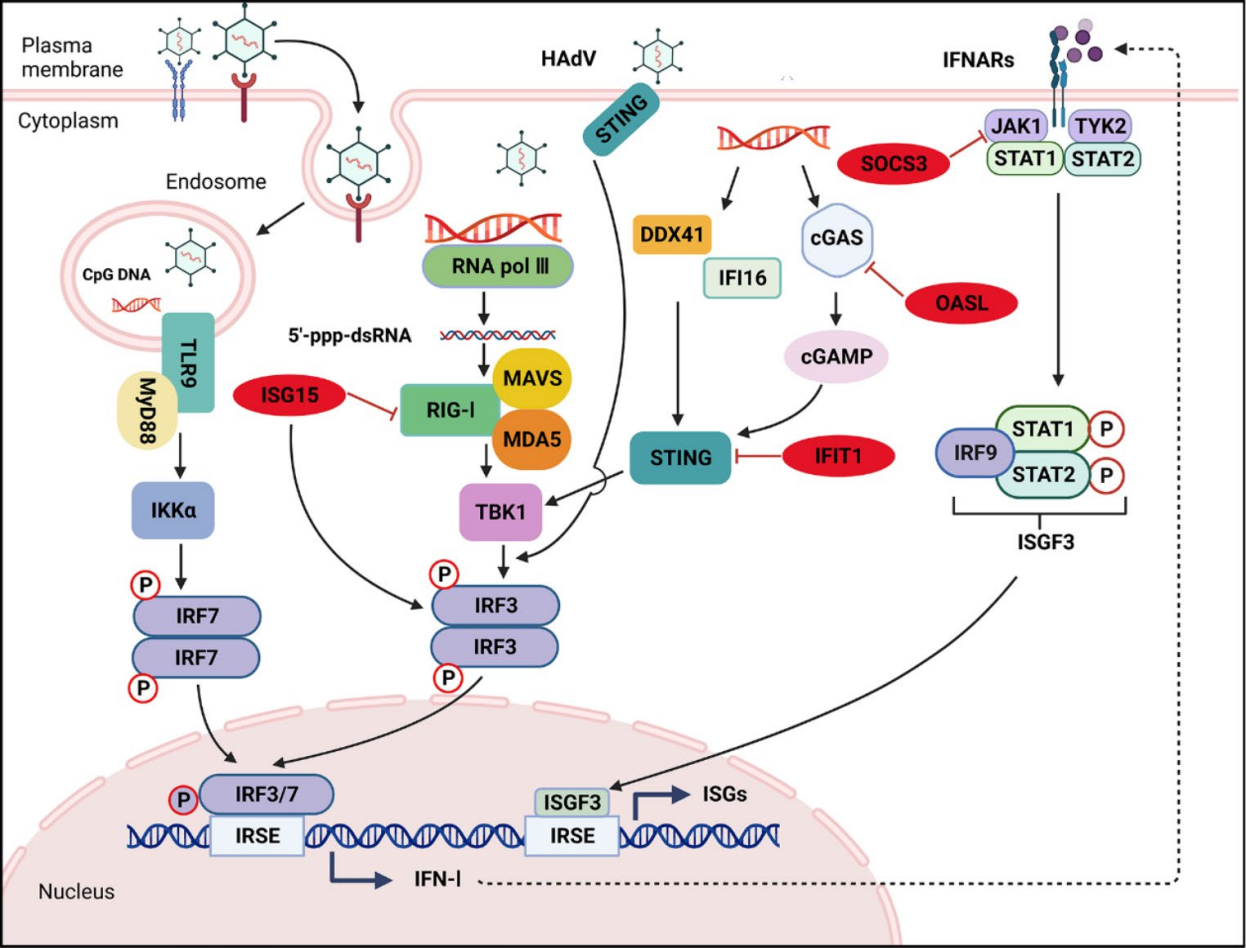
Antiviral innate immune signaling pathways in adenovirus infection and interaction between adenovirus and host cells. (cGAMP, cyclic GMP-AMP; cGAS, cyclic GMP-AMP synthase; DDX41, DEADV-box polypeptide 41; HAdV, human adenovirus; IFI16, interferon gamma-inducible protein 16; IFIT1, IFN-induced protein with tetratricopeptide repeats 1; IFN-I, type I interferon; IFNARs, type I interferon receptors; IRF3, interferon regulatory factor 3; IRF7, interferon regulatory factor 7; IRF9, interferon regulatory factor 9; IRSE, IFN-response elements; ISG15, IFN-stimulated gene product 15; ISGF3, interferon-stimulated gene factor 3; ISGs, interferon-stimulated genes; JAK1, Janus kinase-1; MAVS, mitochondrial antiviral-signaling protein; MDA5, melanoma differentiation-associated gene 5; MyD88, myeloid differentiation primary response gene 88; OASL, oligoadenylate synthetases-like; RIG-I, retinoic acid-inducible gene I; STAT1, signal transducer and activator of transcription 1; STAT2, signal transducer and activator of transcription 2; STING, stimulator of interferon genes; TBK1, TANK-binding kinase 1; TLR9, Toll-like receptor 9; TYK2, tyrosine kinase 2). Created with BioRender.com

Transcriptome sequencing showed that the expression levels of *SOCS3*, *OASL*, *ISG15*, and *IFIT1* increased significantly over time, especially at 48 hpi (except *SOCS3*) and 72 hpi (Fig. 7A), suggesting that these genes might play an important role in adenovirus infection. Furthermore, there were expression correlations among the four genes (*p* < 0.05) (excluding *SOCS3* vs. *ISG15* and *SOCS3* vs. *IFIT1*) (Fig. 7B), demonstrating a specific interaction between candidate genes. We used the online software GeneMANIA (http://genemania.org/, accessed on 5 January 2021) to construct a gene–gene interaction network. Besides the four candidate genes, the network included another 20 potentially frequently interacting genes and 1122 links (interactions), and most of them were associated with regulation of viral process and response to interferon (Fig. 7C). The innate immune system provides a robust first line of defense against viral infection, and IFN-I is considered a key component of antiviral innate immunity. Upon invasion of the host, a complex and intense battle occurs between host cells and viruses. The virus may evade the host’s immune response by inducing the synthesis of molecules that inhibit interferon signaling [33], with the involvement of SOCS3, OASL, ISG15, and IFIT1. Based on previous literature and our analysis, we propose the following model. Adenovirus binds to its receptor and enters the target cell. Toll-like receptor 9 (TLR9), the only known endosomal localized DNA sensor that can detect unmethylated viral DNA containing cytosine-phosphate-guanosine (CpG) motifs, recruits myeloid differentiation primary response gene 88 (MyD88), leading to activation of IRF7 and NF-κB, which induce IFN-I and various chemokines and cytokines [39]. The viral genome can be sensed by RNA polymerase III, IFN gamma-inducible protein 16 (IFI16), DEAD-box helicase 41 (DDX41), cyclic GMP-AMP (cGAMP) synthase (cGAS), and several other DNA sensors, of which RNA pol III transcribes dsDNA into 5’-triphosphate double-stranded RNA (5’-ppp-dsRNA). Once dsRNA binds to retinoic-acid-inducible gene I (RIG-1) and melanoma differentiation-associated gene 5 (MDA5), the antiviral RIG-I-mitochondrial antiviral-signaling protein (MAVS) and MDA5-MAVS signaling pathways are activated [46]. cGAS, IFI16, and other DNA sensors then activate the adaptor protein stimulator of IFN genes (STING), which further recruit and phosphorylate TANK-binding kinase 1 (TBK1) to relay signals to IRF3 to induce the production of IFN-I [39, 46]. IFN-I interacts with its universally expressed receptors (IFNARs) and successively phosphorylates signal transducer and transcription activator (STAT) family proteins through Janus protein kinase (JAK) family members. The phosphorylated STAT1/STAT2 heterodimer associates with IRF9 to form the transcriptional factor complex IFN-stimulated gene factor 3 (ISGF3), which translocates to the nucleus and binds the IFN-response elements (IRSE) in ISG promoters to induce the expression of ISG products, including OASL, ISG15, and IFIT1 [31]. The ISGs exert complex functions in adenovirus infection. For instance, IFIT1 can disrupt viral DNA replication [7] and translation [42] and dampen virus-induced innate immune signaling by binding to STING [7]. ISG15 downregulates RIG-I-mediated signaling to reduce IFN promoter activity but also inhibits viral replication by sustaining IRF3 activation [26]. IFN induction and signaling induce OASL, which then binds to and inactivates cGAS to negatively regulate IFN production [19]. SOCS3 binds to JAKs to inhibit their activity and ubiquitinates and degrades JAKs via the SOCS box, thereby inhibiting JAK/STAT signaling (Fig. 8) [13, 24].

## Discussion

HAdVs are a frequent cause of severe pediatric pneumonia, with HAdV-7 in particular causing more-severe clinical consequences, which appear to be associated with the high replication competence of HAdV-7 in human lung [3] and prolonged virus shedding and persistence in infected cells. Some possible mechanisms have been discussed previously. For example, adenovirus proteins such as E1A may suppress type I interferon signaling, but the pathophysiological mechanisms involved in HAdV-7 infection are complex and remain unclear. Viral infection usually results in changes in the expression of host genes following replication. Thus, host cell transcriptomes can reflect the changes in expression of specific genes and pathways that occur during infection [15, 22]. WGCNA is an effective method of mining data to analyze complex genetic networks in HAdV-7 infection. One of the advantages of WGCNA is its high reliability and biological significance, because the analysis focuses on the association between coexpression modules and infection traits [4]. In this study, using the gene expression data obtained by RNA-Seq from HAdV-7-infected and mock-infected A549 cells, a total of 12 coexpression modules were constructed using WGCNA. Of these, the blue, tan, and brown modules were positively correlated with traits of HAdV-7-infected cells at 24, 48, and 72 hpi, respectively. Genes in the same module are considered functionally related to each other. Functional enrichment analysis indicated that genes in the blue module were primarily enriched in cell cycle regulation and DNA replication, consistent with previous studies showing that adenovirus infection has also proceeded far into the early phase at 24 h and expression changes favor its DNA replication, with about 50% of those genes with known functions involved in cell cycle control [43]. Genes in the tan module were significantly associated with cell metabolism. At this time point, the virus has gained control of the cellular metabolic machinery to create conditions under which the viral genome can replicate efficiently [44]. Genes in the brown module were mainly enriched in mitochondrial respiratory chain functions, protein translation, and apoptosis. During this period, considerable energy and proteins are needed to complete virus assembly. Moreover, cell death is thought to be imminent, facilitating efficient release and spread of the viral progeny. DEGs with high intramodular connectivity were identified as hub genes for HAdV-7 infection. Furthermore, concordant genes among hub genes from the blue, tan, and brown modules and DEGs in the GSE68004 dataset were identified using a Venn diagram (Figs. 3-5C). Of these, *SOCS3*, *OASL*, *ISG15*, and *IFIT1* were found to be involved in the process of adenovirus infection and were thus regarded as candidate genes. During viral invasion, the host rapidly establishes several defensive mechanisms by initiating the innate immune response. Conventionally, IFN-I is the major component of the innate immune system against viral infection via induction of various ISGs [20, 26]. *OASL*, *ISG15*, and *IFIT1* are ISGs that are involved in interactions between adenoviruses and host cells (Fig. 8). *OASL* codes for an important ISG and plays different roles in DNA and RNA viruses by inhibiting cGAS-mediated IFN production and enhancing RIG-I-mediated IFN induction, respectively [5, 19]. Several studies have shown that OASL inhibits the replication of some RNA viruses, such as vesicular stomatitis virus (VSV), Sendai virus (SeV), and respiratory syncytial virus (RSV) [5, 48, 49]. Compared to RNA viruses, however, much less is known about the effects of OASL in the context of DNA virus infection. Human OASL and mouse Oasl2 have been reported to promote the replication of certain DNA viruses, including herpes simplex virus (HSV), mouse cytomegalovirus (MCMV), and adenovirus [10]. Therefore, OASL may serve as a biomarker for discrimination between DNA and RNA virus infection. As one of the most highly upregulated genes during viral infections, *ISG15* acts as both an effector and a signaling molecule in various phases of the innate immune response [8]. ISG15 is involved in many antiviral signaling pathways, both intracellular and extracellular, and activates various immune cells and promotes the production of many antiviral cytokines to facilitate viral clearance [8, 26]. Through the study of *ISG15*-deficient patients, it was revealed that human *ISG15* is redundant for antiviral immunity and negatively regulates IFN-I immunity [42]. Although the multiple biological functions of ISG15, including as a biomarker of antiviral treatment [14], offer promise for intervention in disease progression, several important questions remain to be answered in future research. The expression of IFIT1 is strongly induced by IFN-I, double-stranded RNAs, and viral infection [28], and increasing evidence has demonstrated that IFIT1 has antiviral activity during both DNA and RNA virus infection, mainly by intervening in translation by differentially recognizing the 5’ terminus of target RNA [30], and it also inhibits the interferon signaling pathway [7]. As one of the best-studied members of the SOCS family, SOCS3 can be stimulated by JAK/STAT signaling to regulate the proinflammatory response via negatively regulating cytokine receptors [21]. There is considerable evidence that multiple viruses can upregulate SOCS3 expression and dampen the host antiviral responses to promote viral replication through various immune evasion strategies [13]. For example, influenza A virus [25], HSV [38], and RSV [47] can suppresses IFN-I production and response by stimulating SOCS3 expression [2], consistent with our data showing that SOCS3 was upregulated after adenovirus infection. SOCS3, a viral virulence factor, may also have therapeutic potential. For example, SOCS3 expression could be manipulated to restore antiviral immune responses. In addition, SOCS3 antagonists have shown antiviral effects on a broad range of viruses in cell and animal models [13, 16, 24].

The data show not only that SOCS3, OASL, ISG15, and IFIT1 are involved in interactions between adenovirus and host cells, together with other molecules that disrupt type I interferon signaling, but also that this activity correlates with their expression levels. We therefore speculate that HAdV-7 inhibits interferon production through multiple targets after infecting host cells, which could partly explain why this virus is associated with especially severe disease and significant morbidity. The present study is the first to investigate coexpression gene networks associated with HAdV-7 infection using WGCNA, but it has several limitations. For instance, we only used a single cell line rather than human specimens, and thus, we selected data obtained from whole blood of children from a public database for validation of hub genes. We also did not study the exact mechanism of the identified key genes in HAdV-7 infection. Further studies are required to evaluate and confirm the involvement of the candidate genes in different races and samples.

## Electronic supplementary material

Below is the link to the electronic supplementary material

Supplementary Material 1

## Data Availability

The datasets generated and/or analysed during the current study are available from the corresponding author on reasonable request.
